# Editorial: Digital technologies in hepatology: diagnosis, treatment, and epidemiological insights

**DOI:** 10.3389/fmed.2026.1850563

**Published:** 2026-05-07

**Authors:** Chenyang Huang

**Affiliations:** People's Liberation Army 989 Hospital, Luoyang, China

**Keywords:** digital hepatology, liver cirrhosis, machine learning, nomogram, predictive modeling

## Aim and scope of the Research Topic

The field of hepatology stands at a pivotal juncture, propelled by the convergence of clinical medicine with the transformative power of digital technologies. The advent of artificial intelligence (AI), big data analytics, and advanced computational methods is reshaping our approach to liver diseases, from understanding population-wide trends to personalizing care at the bedside. This Research Topic, “*Advances in Digital Hepatology*,” was conceived to capture and catalyze this evolution. Our aim was to curate a collection of research that critically examines and demonstrates how digital tools—encompassing AI, machine learning (ML), advanced data processing, and novel diagnostic technologies—can be leveraged to enhance the prevention, diagnosis, treatment, and epidemiological understanding of hepatobiliary diseases. We sought contributions that not only showcase technological innovation but also address the persistent challenges in early diagnosis, risk stratification, and the development of tailored therapeutic strategies, ultimately paving the way for more precise and equitable liver healthcare.

## The contributions in context

The 20 articles published in this Topic collectively paint a comprehensive picture of the current landscape, aligning with and expanding upon our initial themes. They can be thematically synthesized into several key strands that reflect the integrative and interdisciplinary nature of modern digital hepatology.

1. Harnessing data for precision diagnosis and prognostic modeling

A core thrust of digital hepatology is the development of tools that move beyond traditional diagnostics toward quantitative, predictive analytics. Several studies exemplify this by constructing and validating AI/ML-driven nomograms. For instance, Zhang et al. and Wang D. et al. developed nomograms to predict in-hospital mortality in cirrhotic patients with sepsis and post-hepatectomy liver failure, respectively. These models integrate clinical and laboratory parameters to provide clinicians with visual, individualized risk assessments. Similarly, Zhou et al. created a predictive model for advanced hepatic fibrosis in Wilson's disease, demonstrating the power of data integration for long-term risk stratification in chronic liver conditions. These works directly address the Theme of “Big data and precision medicine,” translating complex patient data into actionable clinical tools.

2. Innovating with novel diagnostic and monitoring technologies

Beyond predictive modeling, this Topic highlights cutting-edge non-invasive diagnostic modalities. Zhaivoronok et al. provided robust evidence for ultrasound-based attenuation coefficient measurement (ACM) as a reliable and cost-effective tool for quantifying hepatic steatosis, validating it against MRI-PDFF. In a review, Gao et al. comprehensively surveyed the rise of non-invasive techniques like transient elastography for diagnosing portal hypertension, underscoring a paradigm shift away from invasive procedures. Further pushing the technological frontier, Pérez-Gómez et al. integrated near-infrared spectroscopy with clinical data using ML for HCV detection, proposing a novel, rapid screening alternative. These contributions vividly illustrate the “Digital liver disease diagnostics and treatment technologies” theme, showcasing tools that enhance accuracy, accessibility, and patient comfort.

3. Deciphering disease mechanisms and risks through data integration

Digital technologies empower a deeper dive into disease etiology and personalized risk factors. Wang Q. et al. employed an integrative transcriptome-wide analysis to identify novel genetic susceptibility loci for alcohol-associated liver disease, bridging genomics with potential therapeutic targets. Wei et al. provided epidemiological evidence from a large cohort linking metabolic dysfunction-associated fatty liver disease (MAFLD) to an increased risk of gallstone incidence, highlighting an important comorbidity pattern. Furthermore, Chen et al. pioneered a multi-platform integration model, combining histopathological image features with multi-omics data to predict molecular subtypes and prognosis in hepatocellular carcinoma (HCC). This study is a prime example of the “Integrative interdisciplinary approaches” theme, merging pathology, genomics, and bioinformatics.

4. Understanding population burden and early pathological shifts

Epidemiology, supercharged by global data resources, provides the essential population context. Yang et al. leveraged the Global Burden of Disease Study 2021 to detail the evolving epidemiology of liver cancer in adolescents and young adults, projecting future burdens and revealing persistent gender and regional disparities. This work directly fulfills the “Epidemiology of liver diseases” and “Application of digital tools in epidemiological research” themes. On a subtler physiological level, Zhu et al. used body composition analysis to identify factors influencing subclinical fluid retention in tumor patients, showcasing how digital phenotyping can detect early, clinically silent pathological changes.

## Synthesis and forward look

The collection of work in this Research Topic demonstrates that digital hepatology is no longer a speculative future but an active, present reality driving progress across the clinical and research spectrum. From global disease surveillance (Yang et al.) to subcellular genetic discovery (Wang Q. et al.), and from point-of-care diagnostic tools (Zhaivoronok et al.) to complex, integrated prognostic systems (Chen et al.), the studies collectively argue for a data-centric, technology-enhanced approach.

Key cross-cutting insights emerge. First, the integration of diverse data types—clinical, imaging, genomic, epidemiological—consistently yields superior predictive power and biological insight compared to single-modal analyses. Second, there is a clear trend toward developing practical, often non-invasive, tools designed for clinical translation and early intervention. Third, these advancements hold promise for reducing disparities by enabling more accessible screening and personalized risk assessment, though the persistent inequalities highlighted in global epidemiology remind us that equitable implementation remains a critical challenge.

Looking ahead, the path forward involves several critical steps: the external validation and real-world clinical integration of the proposed AI/ML models; the continued refinement of non-invasive biomarkers; the ethical and standardized application of big data; and fostering deeper collaborations between clinicians, data scientists, and bioengineers. We hope this Research Topic serves as a valuable resource and inspiration, accelerating the journey toward a future where digital technologies are seamlessly woven into the fabric of hepatology, ensuring earlier, more precise, and more personalized care for all patients with liver disease.

